# Methanol fermentation increases the production of NAD(P)H-dependent chemicals in synthetic methylotrophic *Escherichia coli*

**DOI:** 10.1186/s13068-019-1356-4

**Published:** 2019-01-21

**Authors:** Xin Wang, Xuelin Wang, Xiaolu Lu, Chen Ma, Kequan Chen, Pingkai Ouyang

**Affiliations:** State Key Laboratory of Materials-Oriented Chemical Engineering, College of Biotechnology and Pharmaceutical Engineering, Nanjing Tech University, Nanjing, 211816 Jiangsu China

**Keywords:** Methanol, Synthetic methylotrophic *E. coli*, Cofactor regeneration, NAD(P)H-dependent chemicals

## Abstract

**Background:**

Methanol has attracted increased attention as a non-food alternative carbon source to sugar for biological production of chemicals and fuels. Moreover, the high degree of reduction of methanol offers some advantages in increasing the production yields of NAD(P)H-dependent metabolites. Here, we demonstrate an example of methanol bioconversion with the aim of improving production of NAD(P)H-dependent chemicals in synthetic methylotrophic *Escherichia coli*.

**Results:**

A synthetic methylotrophic *E. coli* was engineered with a nicotinamide adenine dinucleotide (NAD^+^)-dependent methanol dehydrogenase (MDH) and ribulose monophosphate (RuMP) pathway. Regarding the limited MDH activity, the role of activator proteins in vivo was investigated, and the NudF protein was identified capable of improving MDH activity and triggering increased methanol metabolism. Using ^13^C-methanol-labeling experiments, we confirmed methanol assimilation in the methylotrophic *E. coli*. A cycling RuMP pathway for methanol assimilation was also demonstrated by detecting multiple labeled carbons for several compounds. Finally, using the NAD(P)H-dependent metabolite lysine as a test, the potential of methanol bioconversion to generate value-added metabolites was determined. To further characterize the benefit of methanol as the carbon source, extra NADH from methanol oxidation was engineered to generate NADPH to improve lysine biosynthesis by expression of the POS5 gene from *Saccharomyces cerevisiae*, which resulted in a twofold improvement of lysine production. Moreover, this new sink further pulled upstream methanol utilization.

**Conclusion:**

Through engineering methanol metabolism, lysine biosynthesis, and NADPH regeneration pathway from NADH, the bioconversion of methanol to improve chemical synthesis was successfully achieved in methylotrophic *E. coli*.

**Electronic supplementary material:**

The online version of this article (10.1186/s13068-019-1356-4) contains supplementary material, which is available to authorized users.

## Background

Microbial production of chemicals and biofuels from feedstock that are both inexpensive and abundant, such as natural gas, offers sustainable and economically attractive alternatives to traditional fermentation processes [1–3]. Given advances in methods of converting natural gas to methanol, methanol has attracted increased attention as a non-food alternative carbon source to sugar in microbial production processes [4–6]. Meanwhile, as the higher reduction degree of methanol than most lignocellulosic sugars, it might be used to enhance the production of some reductive products, such as alcohols, carboxylic acids, and fatty acids when used alone or as a co-substrate.

Methylotrophs represent a group of organisms that use methane or methanol as carbon and energy sources to produce metabolites, including biofuels and chemicals. Efforts to engineer native methylotrophs (e.g., *Bacillus methanolicus*, and *Methylobacterium extorquens*) [7–9] have been hampered due to the inefficient genetic tools. Developing synthetic methylotrophy using platform organisms, such as *Corynebacterium glutamicum* and *E. coli*, has become an increasingly attractive possibility for methanol bioconversion [10–12].

In aerobic methylotrophs, methanol is initially oxidized to formaldehyde by methanol dehydrogenase (MDH). Formaldehyde is subsequently assimilated for energy generation via the serine pathway, the ribulose monophosphate (RuMP) pathway, or the ribulose bisphosphate (RuBP) pathway [5]. The MDHs can be divided into three classes based on their electron acceptor: pyrroloquinoline quinone (PQQ) dependent in Gram-negative bacteria, NAD dependent in Gram-positive bacteria, and oxygen dependent in methylotrophic yeasts, respectively [5, 13, 14]. As a favorable option for synthetic methylotrophy [5], electrons, derived from methanol oxidation that was catalyzed by NAD^+^-dependent MDHs, are stored in NADH, which can be used to improve production of target metabolites without sacrificing additional carbons. For the formaldehyde assimilation process, the RuMP pathway, which fixes formaldehyde to the pentose phosphate pathway (PPP) intermediate ribulose-5-phosphate via the two core enzymes, including 3-hexulose-6-phosphate synthase (HPS) and 6-phospho-3-hexuloisomerase (PHI) [8], has been shown to be more bioenergetically favorable in terms of ATP generation.

*Escherichia coli* is an important platform organism that has been extensively engineered for superior industrial production of an enormous range of useful metabolites. In the past few years, several efforts to engineer improved methanol utilization ability in *E. coli* have been made. For example, in ^13^C-methanol-labeling experiments, ^13^C-labeled glycolytic intermediates were detected in synthetic methylotrophic *E. coli* [12]. Using MDH from *B. stearothermophilus* and the RuMP pathway from *B. methanolicus*, 30% improvement in biomass was observed in a methylotrophic *E. coli* strain and methanol-derived naringenin production [15]. Bennett et al. [16] further improved methanol assimilation by expressing the nonoxidative pentose phosphate pathway (PPP) from *B. methanolicus*. However, the effect of methanol consumption on intracellular cofactor levels, such as NADH and NADPH, was rarely characterized in synthetic methylotrophy, as well as the use of excess electrons from MeOH consumption to improve the yields of desirable metabolites.

In the present study, a synthetic methylotrophic *E. coli* was engineered by employing the NAD^+^-dependent MDH and RuMP pathways. Two MDH activator proteins were then coexpressed to analyze their role in regulating methanol metabolism in vivo, and the protein NudF was found capable of improving methanol metabolism. After optimizing the culture condition, ^13^C-methanol-labeling experiments were carried out to confirm the methanol assimilation in methylotrophic *E. coli.* Finally, with lysine as an example, the possibility that bioconversion of methanol to generate value-added metabolites was determined. The extra NADH from methanol oxidation was also engineered as an alternative way to generate NADPH for improving biosynthesis of the desired metabolites.

## Results

### The construction of synthetic methylotrophy *E. coli* for methanol utilization

**Fig. 1:**
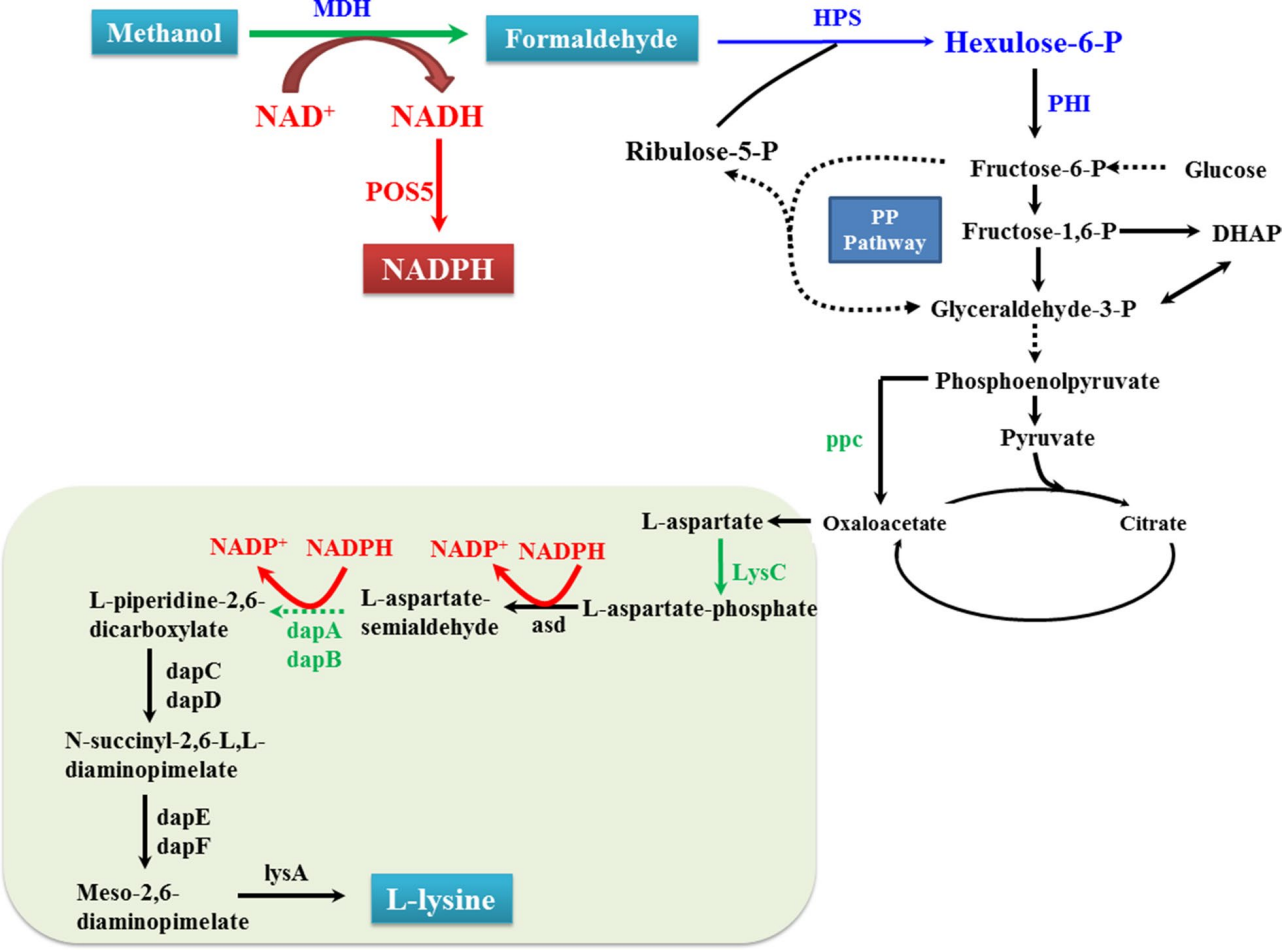
Methanol bioconversion for improved lysine synthesis in synthetic methylotrophic *E. coli*. Enzymes required for the assimilation of methanol into central metabolism are shown in blue: *MDH* methanol dehydrogenase, *HPS* 3-hexulose-6-phosphate synthase, and *PHI* 6-phospho-3-hexuloisomerase. The genes overexpressed in the lysine biosynthetic pathway are shown in green. The cofactor generation pathway was reconstructed by expressing *POS5* from *S. cerevisiae* to convert extra NADH and generate NADPH, which is shown in red

Due to the higher generations of ATP and NAD(P)H, we considered the NAD-dependent MDH and -RuMP pathways being most favorable for engineering synthetic methylotrophy [5, 12, 15]. Enzymes with high catalytic activity are prerequisites for efficient engineering of the desired phenotype, and *Mdh2*, *Hps* and *Phi* from *B. methanolicus* had previously been determined to be the most effective [12]. Based on these previous results, the methanol metabolic pathway was assembled in *E. coli* BL21(DE3) (Fig. 1).

**Fig. 2:**
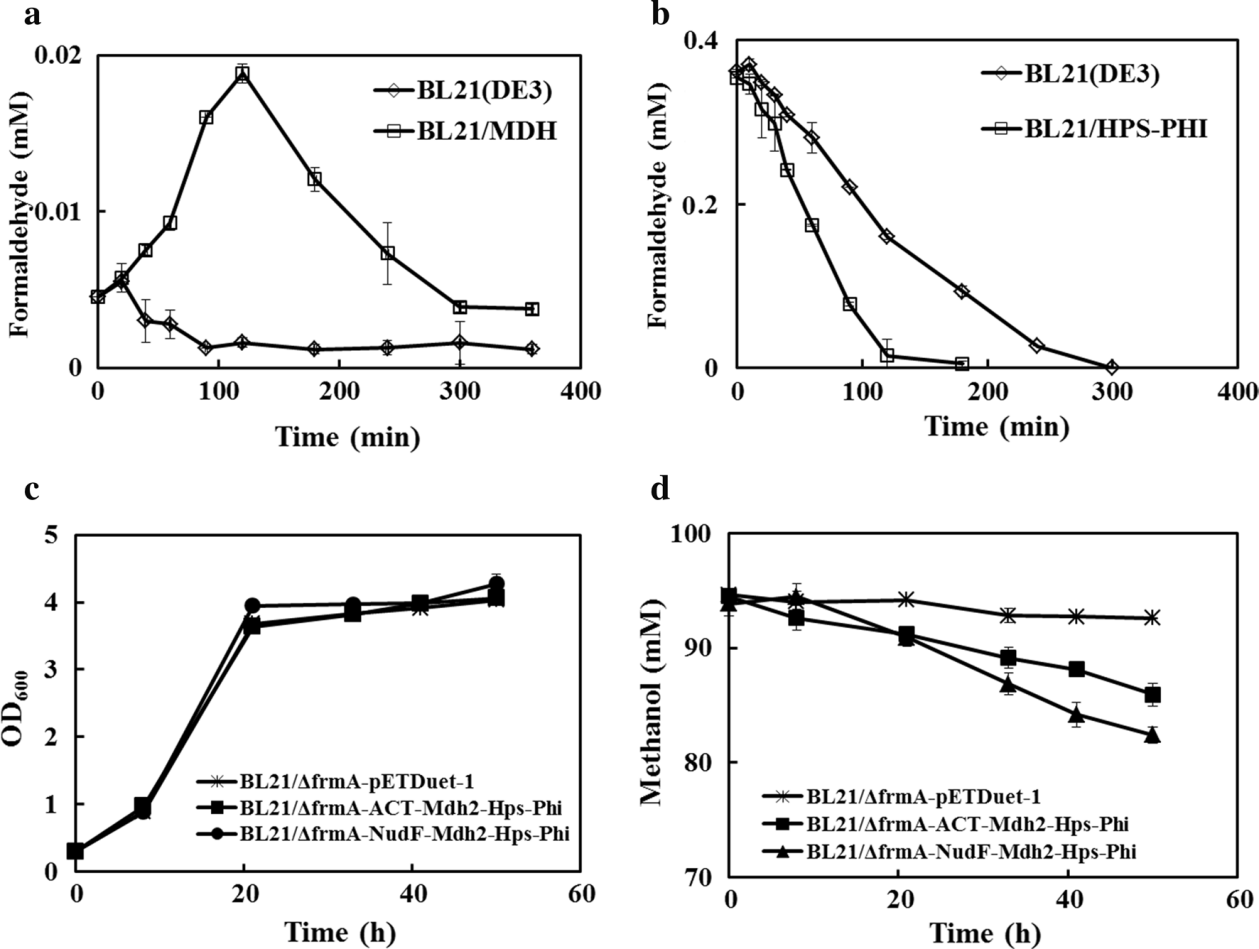
The characterization of the synthetic *E. coli*. **a** Activity of MDH from *Bacillus methanolicus* MGA3 and an empty vector control in vivo were evaluated by analyzing the formaldehyde production in *E. coli* BL21(DE3) in M9 minimal media supplemented with 100 mM methanol. **b** Activity of HPS-PHI operon from *Bacillus methanolicus MGA3* and empty vector control in vivo were assayed by analyzing the formaldehyde consumption in *E. coli* BL21(DE3) in M9 minimal media supplemented with 100 mM methanol. **c** The growths of the strains BL21/ΔfrmA-Mdh2-Hps-Phi, BL21/ΔfrmA-ACT-Mdh2-Hps-Phi, and BL21/ΔfrmA-NudF-Mdh2-Hps-Phi in M9 medium supplemented with 55 mM glucose and 100 mM methanol. **d** Methanol consumption by the strains BL21/ΔfrmA-Mdh2-Hps-Phi, BL21/ΔfrmA-ACT-Mdh2-Hps-Phi, and BL21/ΔfrmA-NudF-Mdh2-Hps-Phi in M9 medium supplemented with 55 mM glucose and 100 mM methanol. Error bars indicate standard error of the mean (*n* = 3)

We then analyzed the activities of MDH and HPS-PHI to identify whether the enzymes for methanol metabolism were functionally produced and activated in *E. coli* BL21(DE3). The MDH activity in *E. coli* BL21(DE3) was evaluated by the measurement of formaldehyde accumulated. After 120 min, the formaldehyde accumulation was successfully detected (Fig. 2a), followed by a decrease in formaldehyde concentration, suggesting the activity of endogenous formaldehyde degradation pathway.

To identify the activity of HPS-PHI, formaldehyde degradation of the whole-cells was tested. Formaldehyde was degraded in both the wild-type strain and HPS-PHI-expressing strain, although the HPS-PHI-expressing *E. coli* strain exhibited a higher degree of degradation (Fig. 2b), confirming the activity of HPS-PHI in *E. coli* BL21(DE3). These data indicate that the methylotrophic enzymes required for methanol utilization are functionally expressed in the engineered *E. coli* BL21(DE3) strain. However, the endogenous formaldehyde degradation pathway in wild-type *E. coli* was found to be highly activated. To avoid their effect on methanol fixation efficiency, an *E. coli* mutant strain, *ΔfrmA*, with a deletion in formaldehyde dehydrogenase, was constructed to prevent the endogenous degradation of formaldehyde and used as the host cell in the following experiment.

### The modification of MDH activity by the coexpression of activator protein

The NAD^+^-dependent MDHs from *Bacillus* spp. have lower affinity toward MeOH compared to higher alcohols. It has been reported that in vitro activity of *B. methanolicus* MDH could be increased by the endogenous activator protein ACT [17]. To test whether the ACT from *B. methanolicus* could increase the MDH activity in *E. coli* in vivo, the ACT protein was coexpressed in *E. coli* BL21(DE3). The presence of ACT had a positive effect on the in vivo activity of MDH in *E. coli*, which was increased to 29.1 mU/mg from 22.1 mU/mg (Table 1).

**Table 1:** The effect of activator proteins in in vivo activities of MDH in *E. coli* BL21/ΔfrmA

Strain	Activities (mU/mg DCW)
MDH	22.1
ACT-MDH	29.1
NudF-MDH	59.8

ACT belongs to the enzyme family of Nudix hydrolases and uses NAD^+^ as a substrate [18]. Ochsner previously showed that in vitro activity of *B. methanolicus* MDH could also be improved by other ACT-like Nudix hydrolases [18]. As a member of the Nudix hydrolase family, NudF from *E. coli* [17] was overexpressed to test its effect on in vivo activity of MDH in *E. coli* BL21(DE3), which significantly increased MDH activity by 2.1 times compared with that by ACT (Table 1).

To further determine the effect of activator proteins on methanol utilization, the engineered strains BL21/ΔfrmA-Mdh2-Hps-Phi, BL21/ΔfrmA-ACT-Mdh2-Hps-Phi, and BL21/ΔfrmA-NudF-Mdh2-Hps-Phi were cultured in M9 medium supplemented with 55 mM glucose and 100 mM methanol. Little difference in growth was observed in the strains BL21/ΔfrmA-pETDuet-1 and BL21/ΔfrmA-ACT-Mdh2-Hps-Phi, and a slight growth increase was observed in the strain BL21/ΔfrmA-NudF-Mdh2-Hps-Phi (Fig. 2c). After fermenting for 50 h, the strain BL21/ΔfrmA-NudF-Mdh2-Hps-Phi consumed 11.9 mM, while 8.0 mM methanol was consumed by the stain BL21/ΔfrmA-ACT-Mdh2-Hps-Phi (Fig. 2d). These results further indicated the positive role of protein NudF in regulating methanol utilization.

### The optimization of methanol utilization in recombinant strain BL21/ΔfrmA-NudF-Mdh2-Hps-Phi

**Fig. 3:**
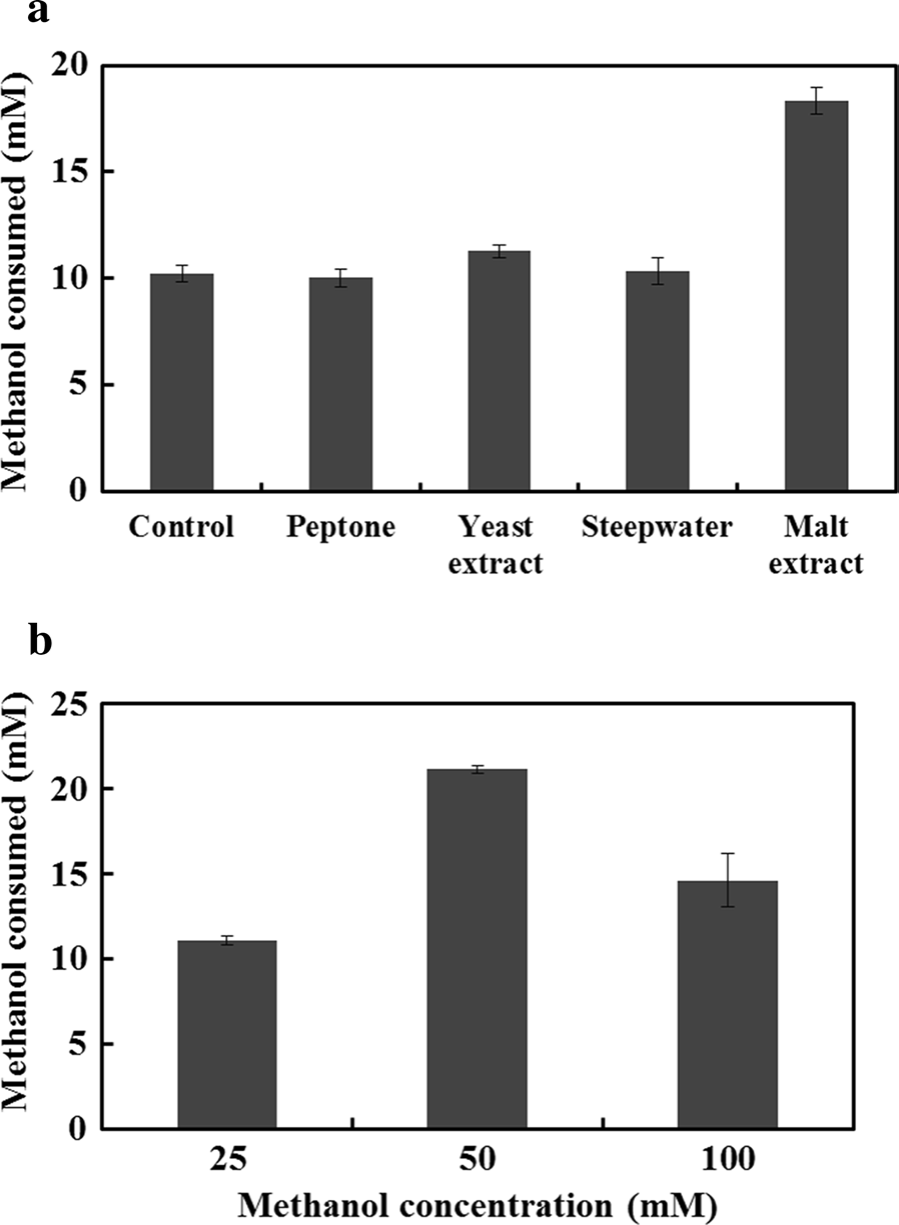
The optimization of methanol metabolism in the synthetic *E. coli* BL21/ΔfrmA-NudF-Mdh2-Hps-Phi. **a** The optimization of a nitrogen source. **b** The optimization of methanol concentration. Error bars indicate standard error of the mean (*n* = 3)

The addition of yeast extract could improve methanol utilization [15]. Given the important role of the nitrogen source, different nitrogen sources were added into the medium to investigate their influence on methanol metabolism of the strain BL21/ΔfrmA-NudF-Mdh2-Hps-Phi (Fig. 3a). Consistent with the previous study [15], the addition of yeast extract moderately improved the methanol utilization from 10.2 mM to 11.3 mM. With regard to the other nitrogen sources, the presence of peptone and steepwater had little effect on methanol metabolism. Notably, the methanol metabolism was largely improved by the addition of malt extract, where 18.4 mM methanol was consumed after fermenting for 50 h. Subsequently, the methanol concentration in the medium was optimized. Under the methanol concentration ranging from 25 to 100 mM, its inhibition on growth was not observed (Additional file 1: Figure S1). A maximum methanol consumption rate was observed when 50 mM of methanol was supplemented (Fig. 3b). After fermenting for 50 h, 21.2 mM of methanol was utilized totally, which was the highest methanol consumption level in synthetic methylotrophic *E. coli* reported to date [12, 15]. Meanwhile, a higher biomass was obtained when methanol was used as a co-substrate (Additional file 1: Figure S1).

## ^13^C-methanol-labeling experiments to identify incorporation of methanol-derived carbon into intracellular metabolites

**Fig. 4:**
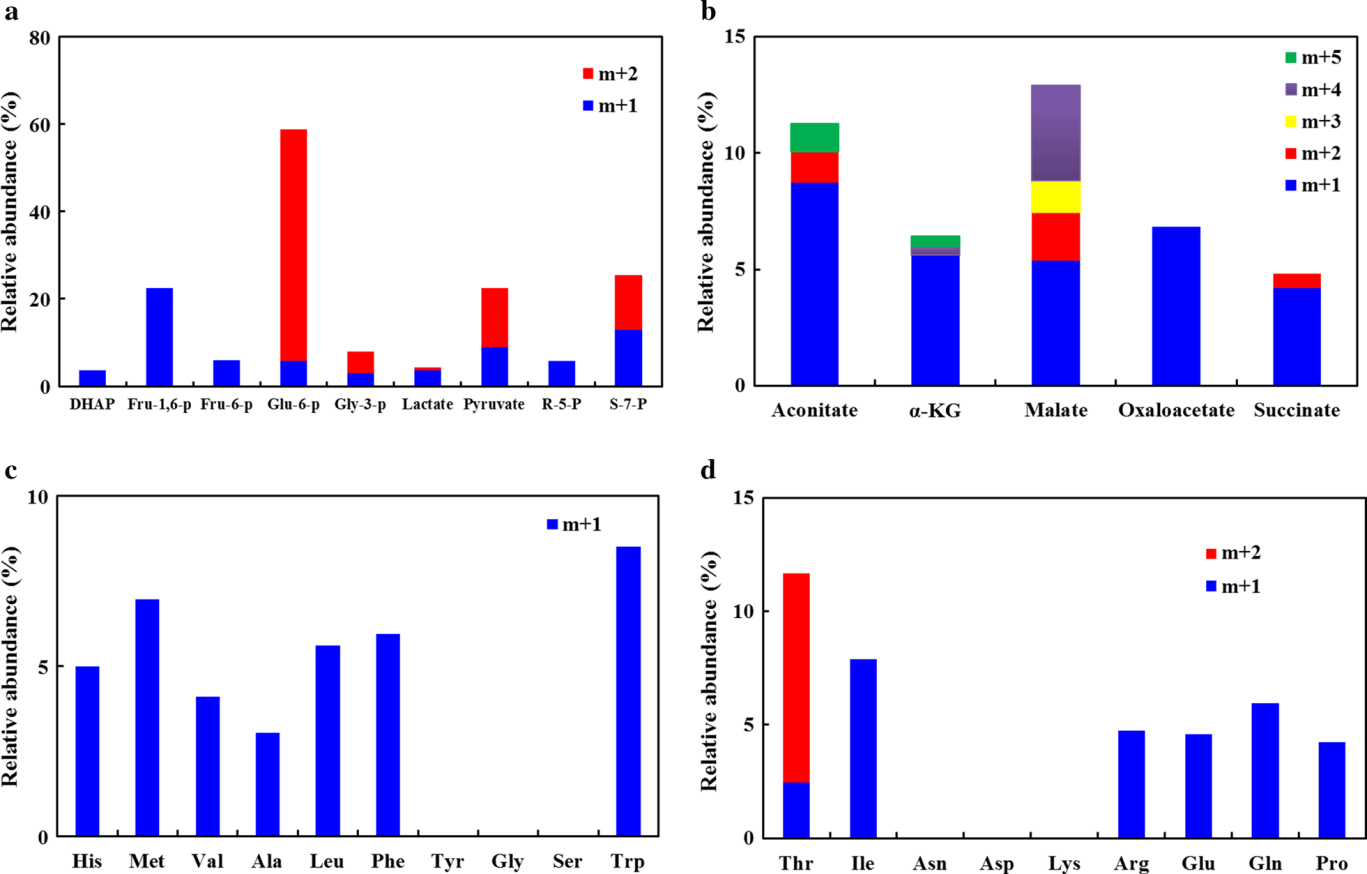
Labeling abundances in different intracellular metabolites from ^13^C-methanol. **a** Relative abundance of mass isotopomers for intermediates involved in glycolysis and phosphate pentose pathway. **b** Relative abundance of mass isotopomers for intermediates involved in the TCA cycle. **c** Relative abundance of mass isotopomers for amino acids derived from the glycolysis pathway. **d** Relative abundance of mass isotopomers for amino acids derived from the TCA cycle. Error bars indicate standard error of the mean (*n* = 3)

To confirm methanol assimilation by the recombinant *E. coli* BL21/ΔfrmA-NudF-Mdh-Hps-Phi, ^13^C-methanol-labeling experiments were carried out in M9 medium supplemented with 55 mM glucose and 50 mM ^13^C-methanol. The cells were sampled after about 25 h when the maximum methanol consumption rate was observed. Detected metabolites mainly included the intermediates associated with glycolysis, the pentose phosphate pathway (PPP), and the tricarboxylic acid (TCA) cycle (Fig. 4).

After cultivation for 25 h, 58.7% of Glu-6-p, 3.6% of DHAP, 22.4% of Fru-1,6-p, 8.0% of Gly-3-p, and 22.5% of pyruvate contained labeled carbon that originated from methanol (Fig. 4a). The labeling of TCA intermediates included 6.8% of oxaloacetate, 11.3% of aconitate, 6.4% of α-KG, 12.9% of malate, and 4.8% of succinate from methanol (Fig. 4b). In addition, we also measured 5.8% labeling ribose-5-p and 25.4% labeling sedoheptulose-7-p in the PPP pathway (Fig. 4a). These results showed that carbon from methanol could successfully pass through the glycolysis, TCA cycle, and PPP pathway.

We also detected 19 amino acids, including Ala, Arg, Asp, Glu, Asn, Gln, Gly, His, Ile, Leu, Lys, Met, Phe, Pro, Ser, Thr, Trp, Tyr, and Val, and 13 amino acids were labeled. Among the labeled amino acids, Val, Ala, Leu, Trp, Phe, and Met were derived from the glycolysis intermediates. Thr, Ile, Arg, Glu, Gln, and Pro were the amino acids derived from TCA cycle intermediates. Although the labeling pool of these amino acids was lower than 10%, except for Thr (11.7%) (Fig. 4c, d), their detection indicated that carbon from methanol could be assimilated into metabolic pathways that branched off of central carbon metabolism, and then used for synthesizing important cell components.

In addition, among the detected metabolites, many of them were labeled by multiple carbons. For example, approximately 52.9% of Glu-6-p, 5.0% of Gly-3-p, and 13.6% of pyruvate involved in the glycolysis pathway exhibited M + 2 labeling (Fig. 4a). Also, approximately 1.3% of aconitate contained M + 2 labeling, and 1.2% contained M + 5 labeling (Fig. 4b). For the compound α-ketoglutarate (α-KG), 0.4% contained M + 4 labeling and 0.5% was fully labeled. 2.1% of the malate contained M + 2 labeling, 1.4% contained M + 3 labeling, and 4.1% was fully labeled (Fig. 4b). Similarly, 0.6% of succinate contained M + 2 labeling (Fig. 4b). We also found that approximately 8.6% of Thr contained M + 2 labeling (Fig. 4d). The detection of multiple carbon-labeled metabolites indicated that the RuMP cycle successfully worked in the recombinant *E. coli* BL21/NudF-Mdh2-Hps-Phi.

### The bioconversion of methanol to enhance l-lysine production

Whitaker et al. [15] previously demonstrated in vivo conversion of methanol to naringenin in synthetic *E. coli*. Leßmeier also confirmed that the nonnatural carbon substrate methanol could be converted at least partially to cadaverine as a nonnative product by a recombinant *C. glutamicum* strain [10]. These works show the potential of methanol to act as a carbon source for the production of value-added chemicals in synthetic methylotrophy. As methanol possesses a high degree of reduction, its consumption can improve the intracellular NADH availability used for facilitating the synthesis of some reductive products. Here, we attempted to use the NADH from methanol consumption to generate NADPH to expand the bioconversion of methanol for improving the synthesis of reductive products in synthetic methylotrophic *E. coli*.

**Fig. 5:**
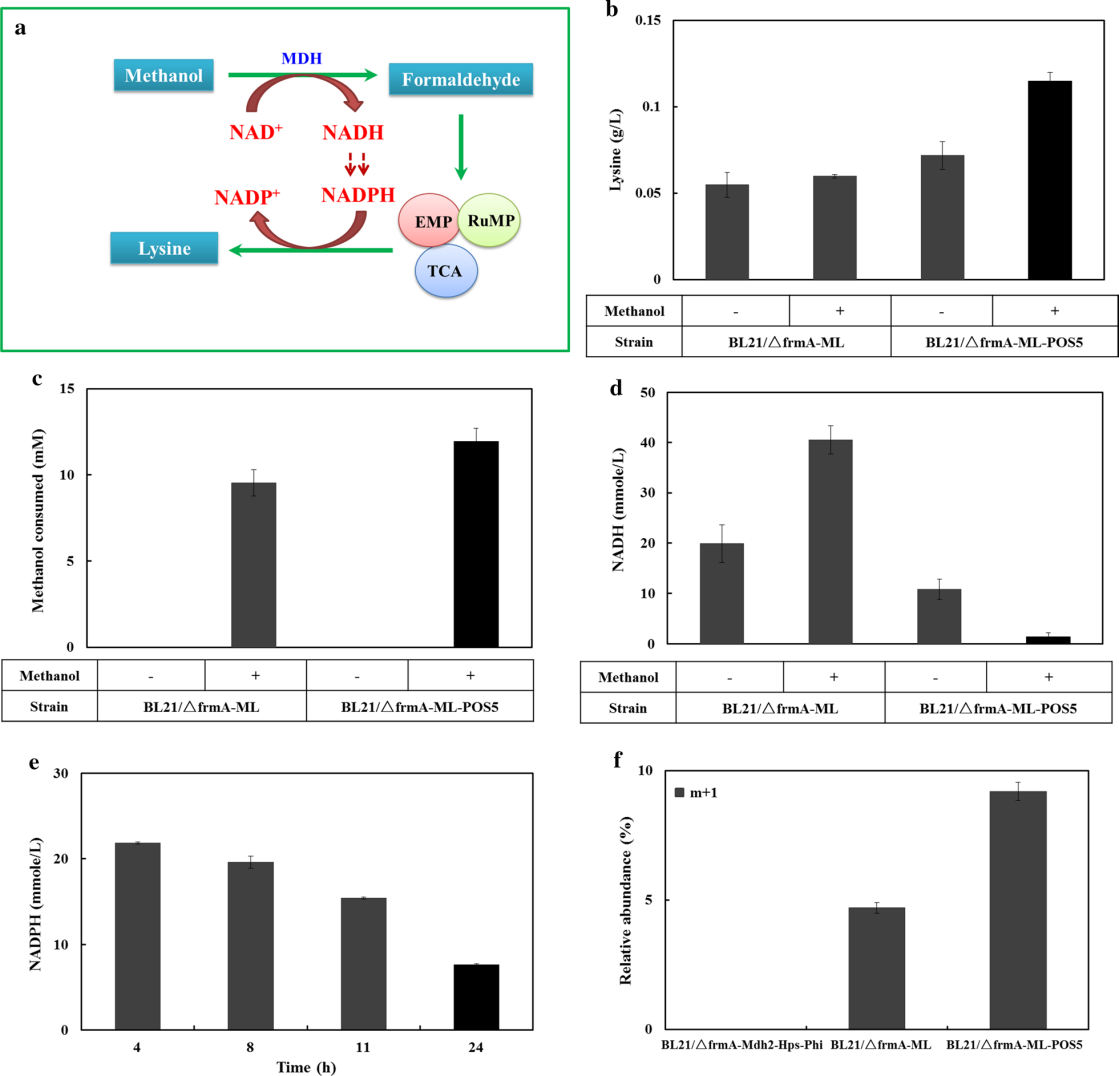
Improved lysine production in synthetic methylotrophic *E. coli*. **a** Methylotrophic *E. coli* was engineered for the bioconversion of methanol to improve lysine production. The extra NADH from methanol consumption was engineered to generate NADPH for lysine production. **b** Lysine production in the strains BL21/ΔfrmA-ML and BL21/ΔfrmA-ML-POS5 cultivated with and without methanol. **c** Methanol consumption in the strains BL21/ΔfrmA-ML and BL21/ΔfrmA-ML-POS5. **d** The intracellular NADH pools in the strains BL21/ΔfrmA-ML and BL21/ΔfrmA-ML-POS5 cultivated with and without methanol. **e** The intracellular NADPH pools in the strains BL21/ΔfrmA-ML-POS5 cultivated with 55 mM glucose and 50 mM methanol. **f** Lysine production from ^13^C-methanol in the strains BL21/ΔfrmA-Mdh2-Hps-Phi, BL21/ΔfrmA-ML, and BL21/ΔfrmA-ML-POS5. Error bars indicate standard error of the mean (*n* = 3)

Amino acids represent one of the largest classes of fermentation products whose syntheses are closely correlated with the availability of NAD(P)H. The synthesis of 1 mol lysine, for example, requires 4 mol NADPH. Several efforts have been made to enhance the supply of NADPH for improved lysine production [19]. Here, we selected lysine as a test case, and engineered the lysine synthetic pathway and NADPH generation pathway from NADH in the recombinant *E. coli* BL21/ΔfrmA-NudF-Mdh2-Hps-Phi (Fig. 5a). As no lysine was labeled from the above ^13^C-methanol-labeling experiment in *E. coli* BL21/ΔfrmA-NudF-Mdh2-Hps-Phi (Fig. 4c), we also focused on whether the carbon flux from methanol could be directed to generate lysine after the reconstruction of the metabolic pathway.

With the recombinant *E. coli* BL21/ΔfrmA-NudF-Mdh2-Hps-Phi, no lysine was detected in the medium. To improve lysine production, the key genes involved in lysine biosynthesis, including *dapA*, *dapB*, *PPC,* and *lysC*^*fbr*^ (a lysine-insensitive aspartokinase) [20, 21], were co-overexpressed in the recombinant *E. coli* BL21/ΔfrmA-NudF-Mdh2-Hps-Phi to generate *E. coli* BL21/ΔfrmA-ML. Thereafter, 0.06 g/L lysine was produced after fermenting for 24 h, with glucose as the sole carbon source (Fig. 5b). Then, methanol was added into the medium to determine its effect on lysine production. After fermenting for 24 h, *E. coli* BL21/ΔfrmA-ML consumed 9.5 mM of methanol (Fig. 5c), while the methanol metabolism significantly increased the intracellular NADH pool (Fig. 5d). However, production of lysine moderately increased in *E. coli* BL21/ΔfrmA-ML when methanol was used as a co-substrate (Fig. 5b).

To explore the potential of methanol metabolism in improving lysine production, we constructed a NADPH-regenerating system from NADH by expressing NADH kinase (Pos5p) from *S. cerevisiae* [22] in *E. coli* BL21/ΔfrmA-ML (Fig. 5a). The intracellular NADH pool was largely decreased when *POS5* was expressed in *E. coli* BL21/ΔfrmA-ML (Fig. 5d). This engineering strategy successfully enhanced lysine production by twofold when methanol was present, while lysine production was only moderately affected in the absence of methanol (Fig. 5b). At the same time, the methanol utilization ability was also improved by the overexpression of *POS5*. With the synthesis of lysine, the NADPH level decreased (Fig. 5e).

Finally, a ^13^C-methanol labeled experiment was carried out to measure ^13^C-labeling patterns of metabolic lysine in recombinant *E. coli* BL21/ΔfrmA-ML, and *E. coli* BL21/ΔfrmA-ML-POS5 (Fig. 5f). No labeled lysine had been detected in *E. coli* BL21/ΔfrmA-Mdh-Hps-Phi. Through engineering the lysine biosynthetic pathway, 4.7% of lysine exhibited M + 1 labeling in the strain BL21/ΔfrmA-ML. With the overexpression of POS5, 9.0% of lysine contained M + 1 labeling. Lysine labeled by multiple carbons was not detected in the recombinant strains.

## Discussion

The bioconversion of methanol has received considerable attention given methanol’s abundance, low price, and high electron and energy content. In recent years, several efforts have been made to engineer *E. coli* as a synthetic methylotrophy [15, 16, 23, 24]. However, little methanol utilization has been demonstrated to date [12, 15]. MDH activity is one of the key factors limiting methanol metabolism efficiency. To address this problem, several strategies—including a scaffoldless strategy to organize MDH, HPS, and PHI into an supramolecular enzyme complex, or screening alternative MDHs with higher levels of activity from other organisms—have been pursued for enhanced methanol utilization in methylotrophic *E. coli* [15, 25]. Here, through the coexpression of the endogenous activator protein ACT, interaction of which with MDH leads to a conformational change to position NAD^+^ and methanol binding sites closer together enabling direct electron transfer [18], the methanol utilization of the methylotrophic *E. coli* was improved. In addition, we found that the methanol metabolism was achieved more efficiently with another endogenous ACT-like Nudix hydrolase, NudF from *E. coli*. Through the coexpression of NudF, the synthetic methylotrophic *E. coli* consumed about 11.9 mM of methanol at a rate of 0.24 mM MeOH/h, the highest value in synthetic methylotrophic *E. coli* reported to date [12, 15]. Prior to that, the highest methanol consumption level in engineered *E. coli* was reported by Whitaker’s group, where the synthetic methyltrophic *E. coli* was constructed by employing a superior NAD-dependent MDH from *B. stearothermophilus* and ribulose monophosphate (RuMP) pathway enzymes from *B. methanolicus,* and 10 mM of methanol was consumed within the fermentation of 72 h [15]. Further engineering the current MDH or sourcing other activator proteins could be performed in the future to optimize methanol utilization in synthetic methylotrophic *E. coli*.

To date, the bioconversion of methanol to high-value chemicals by synthetic methylotrophic *E. coli* was rarely demonstrated [15]. Whitaker et al. [15] successfully measured the production of naringenin through the combination of plasmid-based modules for both methanol assimilation and naringenin biosynthesis. Here, we give another example to broaden the potential of methanol to produce value-added chemicals as the carbon source. That the biosynthesis of most useful metabolites produced by industrial organisms requires electrons in the form of NADH or NADPH is well known. Given the high degree of reduction of MeOH, we selected the compound lysine, biosynthesis of which was closely associated with intracellular NADPH supply, to demonstrate the bioconversion of methanol. Notably, through engineering methanol metabolism, lysine biosynthesis, and NADPH regeneration pathway from NADH, the carbon flux from methanol to lysine was tracked, and the increased lysine production was observed. Our results further show the potential of methanol in the bioconversion of value-added chemicals as a carbon source, and confirmed the potential of methanol to improve biosynthesis of some NAD(P)H-dependent metabolites.

During the methanol bioconversion to lysine, we found that the expression of POS5 for improved lysine biosynthesis could further drive methanol metabolism. The methanol consumption of *E. coli* BL21/ΔfrmA-ML-POS5 was improved by 1.3-fold compared to *E. coli* BL21/ΔfrmA-ML. One of the key obstacles limiting methanol assimilation in synthetic methylotrophic *E. coli* is the thermodynamically challenging oxidation of methanol to formaldehyde with NAD^+^ as electron acceptor. Woolston et al. [23] identified that NADH was a potent kinetic inhibitor of this enzyme. The typical cellular levels of NADH could inhibit MDH activity by ~ 50% [23]. In the present study, the expression of POS5 from *S. cerevisiae* converted the extra NADH to generate NADPH for lysine production, and largely decreased the intracellular NADH level (Fig. 5d), which was of great benefit for improving methanol oxidation. Our wok provides an example how a new sink pulls upstream methanol utilization, which in return increases rate of NAD(P)H-dependent product generation. However, methanol metabolism efficiency was still far below the industrial application level at present, and the use of methanol as the sole carbon source was also a challenge in synthetic methylotrophy. As we observed in this study, the synthetic *E. coli* could not grow on methanol as the sole carbon when glucose was absent. In future studies, improving ribulose-5-phosphate regeneration, and developing MDH variants insensitive to NADH level, would be the focus for the further engineering of synthetic methylotrophic *E. coli* strain.

## Conclusion

A synthetic methylotrophic *E. coli* was engineered using a NAD+-dependent MDH and RuMP pathway. To improve methanol oxidation, the effect of the MDH activator protein was determined in vivo. In addition, the engineered *E. coli* strain was endowed with the ability to convert methanol into value-added metabolites. More importantly, we demonstrated for the first time an example of reconstructing the intracellular cofactor regeneration pathway based on methanol oxidation to improve the synthesis of NADPH-dependent metabolite, and further drive methanol consumption.

## Methods

### Strain and plasmid construction

All the strains and plasmids used in this study are listed in Additional file 1: Table S1. The primers used for plasmids construction are presented in Additional file 1: Table S2. The gene *Mdh2*, *Hps*-*Phi* operon, and *ACT* from *Bacillus methanolicus* MGA3 were codon-optimized and synthesized by Sprin GenBioTech CO., LTD (Nanjing, China), respectively. The *Mdh2* fragment was inserted into *Nco*I/*Bam*HI sites of plasmid pETDuet to yield plasmid pETDuet-Mdh2. The *Hps*-*Phi* operon was inserted into *Nde*I/*Xho*I sites of pETDuet-Mdh2 and pETDuet-1 to generate plasmid pETDuet-Mdh2-Hps-Phi and pETDuet-Hps-Phi, respectively. Then, the gene *NudF* amplified from *E. coli* MG1655 genome with primer 1 and primer 2 was inserted into *Sac*I/*Sal*I sites of pETDuet-Mdh2 and pETDuet-Mdh2-Hps-Phi to generate plasmid pETDuet-NudF-Mdh2 and pETDuet-NudF-Mdh2-Hps-Phi. The fragment of *ACT* replaced the gene *nudF* to generate plasmid pETDuet-ACT-Mdh and pETDuet-ACT-Mdh-Hps-Phi.

The gene *POS5* was amplified from *Saccharomyces cerevisiae* genome with primer 3 and primer 4 and inserted into *Nde*I/*Xho*I sites of pETDuet to generate plasmid pETDuet-*POS5*. Subsequently, the DNA fragment of T7-POS5 amplified from plasmid pETDuet-*POS5* with primer 5 and primer 6 was inserted into *Xho*I site of pETDuet-*ACT*-*Mdh2*-*Hps*-*Phi* to generate plasmid pETDuet-*ACT*-*Mdh2*-*Hps*-*Phi*-*POS5*.

The DNA fragment of *dapA*-*dapB*-*LysC*^*fbr*^ was amplified from plasmid pTrc99a-*dapA*-*dapB*-*LysC*^*fbr*^ constructed in our previous study with primer 7 and primer 8 [26], which was then inserted into *Spe*I/*Nco*I sites of pCWJ-*PPC* to yield the plasmids pCWJ-*dapA*-*dapB*-*LysC*^*fbr*^-*PPC*.

For deleting frmA gene, the plasmid pTarget-frmA, used in frmA modification with a targeting N20 sequence, was constructed and cotransformed to *E. coli* BL21(DE3) with plasmid pCas9 and donor DNA. With the plasmid pTarget as a template, the pTarget-frmA was obtained by inverse PCR with the primer Target-frmA-F and Target-frmA-R and followed by self-ligation. A 1000-bp donor sequence was designed by overlap PCR with the *E. coli* genome as a template.

### Media and growth condition

The *E. coli* strains were cultured in Luri-Bertani (LB) medium which contained 10 g/L tryptone, 5 g/L yeast extract, and 5 g/L NaCl. The M9 medium supplemented with 10 g/L glucose, 0.5 g/L NaCl, 10 g/L NH_4_Cl, 3 g/L KH_2_PO_4_, and 17.1 g/L NaHPO_4_·12H_2_O was used for the analysis of methanol consumption and enzyme assays. Gene expression was induced by 0.05 mM of isopropyl -d-1-thiogalactopyranoside (IPTG). The strain was cultured at 200 rpm and 37 °C. Appropriate antibiotics were added at the following concentrations: ampicillin, 100 μg/mL, chloramphenicol, 34 μg/mL.

For the fermentation, a single colony was inoculated into the LB medium supplemented with 100 μg/mL Amp or 34 μg/mL Cm, and cultivated for 10 h approximately. Then, the cultures were centrifuged, and resuspended in 50 mL M9 medium. The initial OD_600_ was 0.3 and incubated at 37 °C and 200 rpm. After about 3 h, 0.05 mM IPTG and certain concentration of methanol were added. To optimize the methanol utilization, the experiment was carried out at the different methanol concentrations ranging from 50 mM to 200 mM. The effects of nitrogen sources, including peptone, yeast extract, steepwater, and malt extract were also investigated. The concentrations of the nitrogen source was 1 g/L.

### In vivo enzyme assays

The in vivo activity of MDH was assayed by the production of the formaldehyde. Cells were grown over night in LB medium. Subsequently, amount of cells corresponding to an OD_600_ of 1 in a volume of 10 mL was pelleted by centrifugation at 5000×*g* for 5 min and resuspended in 9 mL M9 medium. The experiment was started by the addition of 1 mL 500 mM methanol, and the cultures were kept at 37 °C. Samples were taken at the specific intervals, and 100 μL of the supernatant was mixed with the same volume of Nash reagent to determine the formaldehyde concentration [27]. One unit (U) was defined as the amount of enzyme that is required to produce 1 μmol formaldehyde produced per minute.

The activity of *Hps*-*Phi* operon was assayed by the consumption of the formaldehyde. Cells were prepared as described above. The reaction was started by adding 0.35 mM formaldehyde. One unit (U) was defined as the amount of enzyme that is required to consume 1 μmol formaldehyde per minute.

### Determination of ^13^C-labeled experiments

For the determination of ^13^C-labeled intracellular metabolites, the assimilation of methanol in recombinant *E. coli* strains was monitored by a ^13^C-methanol-labeling experiment. The strain *E. coli* BL21/ΔfrmA-NudF-Mdh-Hps-Phi was cultivated in LB medium supplemented with 100 µg/mL Amp and 0.5 mM IPTG for 10–12 h. Cells were then transferred to M9 medium with 50 mM ^13^C-methanol (Sigma-Aldrich). After the fermentation of 25 h, cells were collected and pretreated as the following steps. Quenching and metabolite extraction were performed according to the methods described previously with minor modifications [27]. The supernatant (800 μL) of metabolite extracts was transferred into a 1.5 mL EP tube, and lyophilizated under low temperature. The dry extracts were then reconstituted in 300 μL of acetonitrile:H_2_O (1:1, v/v), sonicated for 10 min and centrifuged 15 min at 10,000×*g* and 4  °C to remove insoluble debris. The supernatants were transferred to HPLC vials and stored at − 80  °C prior to LC–MS/MS analysis. For the determination of ^13^C-labeled lysine, *E. coli* BL21/ML-POS5 was cultivated, sampled, and treated as the above steps. For each sample, three replicates were carried out.

### Analytical methods

Cell growth was determined by measuring the OD_600_ using a Beckman Coulter DU370 spectrophotometer. Methanol concentrations were measured using an Agilent 1290 Infinity System (Santa Clara, CA, USA) equipped with an Aminex HPX-87H column. l-Lysine was determined using SBA-40E immobilized enzyme biosensor (Shandong, China) [21]. NADH and NADPH were assayed by means of the NAD/NADH quantitation Kit and the NADP/NADPH quantitation Kit (Sigma-Aldrich).

LC–MS/MS analyses were performed using an UHPLC system (1290, Agilent Technologies) equipped with a UPLC BEH Amide column (1.7 μm, 2.1*100 mm, Waters) coupled to Triple TOF 6600 (Q-TOF, AB Sciex). The mobile phase consisted of 25 mM NH_4_OAc and 25 mM NH_4_OH in water (pH = 9.75) (A), and acetonitrile (B) was carried with elution gradient as follows: 0 min-95% B, 0.5 min-95% B, 7 min-65% B, 8 min-40% B, 9 min-40% B, 9.1 min-95%, and B, 12 min-95% B, which was delivered at 0.5 mL/min. The injection volume was 1 μL. The Triple TOF mass spectrometer was used to assess its ability to acquire MS/MS spectra on an information-dependent basis (IDA) during a LC/MS experiment. ESI source conditions were set as follows: Ion source gas 1 as 60, Ion source gas 2 as 60, Curtain gas as 35, source temperature 550 °C, Ion Spray Voltage Floating (ISVF) 5500 V or − 4500 V in positive or negative modes, respectively.

## Additional file

**Additional file 1: Table S1.** Strains and plasmids used in this work. **Table S2.** Primers used in this study. **Figure S1.** The growth of BL21/ΔfrmA-NudF-Mdh2-Hps-Phi under the different concentration of methanol.

## Abbreviations

DHAP: dihydroxyacetone phosphate
α-KG: α-ketoglutarate
His: histidine
Met: methionine
Val: valine
Ala: alanine
Leu: leucine
Phe: phenylalanine
Tyr: tyrosine
Gly: glycine
Ser: serine
Trp: tryptophane
Thr: threonine
Ile: isoleucine
Asn: asparagine
Asp: aspartic acid
Lys: lysine
Arg: argnine
Gl: glutamic acid
Gln: glutamine
Pro: proline

## References

[1] Dürre P, Eikmanns BJ (2015). C1-carbon sources for chemical and fuel production by microbial gas fermentation. Curr Opin Biotechnol.

[2] Humphreys CM, Minton NP (2018). Advances in metabolic engineering in the microbial production of fuels and chemicals from C1 gas. Curr Opin Biotechnol.

[3] Marlow JJ, Kumar A, Enalls BC, Reynard LM, Tuross N, Stephanopoulos G (2018). Harnessing a methane-fueled, sediment-free mixed microbial community for utilization of distributed sources of natural gas. Biotechnol Bioeng.

[4] Bennett RK, Steinberg LM, Chen W, Papoutsakis ET (2018). Engineering the bioconversion of methane and methanol to fuels and chemicals in native and synthetic methylotrophs. Curr Opin Biotechnol.

[5] Whitaker WB, Sandoval NR, Bennett RK, Fast AG, Papoutsakis ET (2015). Synthetic methylotrophy: engineering the production of biofuels and chemicals based on the biology of aerobic methanol utilization. Curr Opin Biotechnol.

[6] Bogorad IW, Chen CT, Theisen MK, Wu TY, Schlenz AR, Lam AT (2014). Building carbon–carbon bonds using a biocatalytic methanol condensation cycle. Proc Natl Acad Sci.

[7] Ochsner AM, Sonntag F, Buchhaupt M, Schrader J, Vorholt JA (2014). Methylobacterium extorquens: methylotrophy and biotechnological applications. Appl Microbiol Biotechnol.

[8] Müller JEN, Heggeset TMB, Wendisch VF, Vorholt JA, Brautaset T (2014). Methylotrophy in the thermophilic *Bacillus methanolicus*, basic insights and application for commodity production from methanol. Appl Microbiol Biotechnol.

[9] Zhu WL, Cui JY, Cui LY, Liang WF, Yang S, Zhang C (2016). Bioconversion of methanol to value-added mevalonate by engineered *Methylobacterium extorquens* AM1 containing an optimized mevalonate pathway. Appl Microbiol Biotechnol.

[10] Leßmeier L, Pfeifenschneider J, Carnicer M, Heux S, Portais JC, Wendisch VF (2015). Production of carbon-13-labeled cadaverine by engineered *Corynebacterium glutamicum* using carbon-13-labeled methanol as co-substrate. Appl Microbiol Biotechnol.

[11] Witthoff S, Schmitz K, Niedenführ S, Nöh K, Noack S, Bott M (2015). Metabolic engineering of *Corynebacterium glutamicum* for methanol metabolism. Appl Environ Microbiol.

[12] Müller JEN, Meyer F, Litsanov B, Kiefer P, Potthoff E, Heux S (2015). Engineering *Escherichia coli* for methanol conversion. Metab Eng.

[13] Krog A, Heggeset TMB, Müller JEN, Kupper CE, Schneider O, Vorholt JA (2013). *Methylotrophic Bacillus* methanolicus encodes two chromosomal and one plasmid born NAD(+) dependent methanol dehydrogenase paralogs with different catalytic and biochemical properties. PLoS ONE.

[14] Yurimoto H, Oku M, Sakai Y (2011). Yeast methylotrophy: metabolism, gene regulation and peroxisome homeostasis. Int J Microbiol.

[15] Whitaker WB, Jones JA, Bennett RK, Gonzalez JE, Vernacchio VR, Collins SM (2017). Engineering the biological conversion of methanol to specialty chemicals in *Escherichia coli*. Metab Eng.

[16] Bennett RK, Gonzalez JE, Whitaker WB, Antoniewicz MR, Papoutsakis ET (2018). Expression of heterologous non-oxidative pentose phosphate pathway from *Bacillus methanolicus* and phosphoglucose isomerase deletion improves methanol assimilation and metabolite production by a synthetic *Escherichia coli* methylotroph. Metab Eng.

[17] Wu TY, Chen CT, Liu JTJ, Bogorad IW, Damoiseaux R, Liao JC (2016). Characterization and evolution of an activator-independent methanol dehydrogenase from *Cupriavidus necator* N-1. Appl Microbiol Biotechnol.

[18] Kloosterman H, Vrijbloed JW, Dijkhuizen L (2002). Molecular, biochemical, and functional characterization of a Nudix hydrolase protein that stimulates the activity of a nicotinoprotein alcohol dehydrogenase. J Biol Chem.

[19] Bommareddy RR, Chen Z, Rappert S, Zeng AP (2014). A de novo NADPH generation pathway for improving lysine production of *Corynebacterium glutamicum* by rational design of the coenzyme specificity of glyceraldehyde 3-phosphate dehydrogenase. Metab Eng.

[20] Xu J, Han M, Ren X, Zhang W (2016). Modification of aspartokinase III and dihydrodipicolinate synthetase increases the production of l-lysine in *Escherichia coli*. Biochem Eng J.

[21] Ying H, Tao S, Wang J, Ma W, Chen K, Wang X (2017). Expanding metabolic pathway for de novo biosynthesis of the chiral pharmaceutical intermediate l-pipecolic acid in *Escherichia coli*. Microb Cell Fact.

[22] Lee WH, Kim JW, Park EH, Han NS, Kim MD, Seo JH (2013). Effects of NADH kinase on NADPH-dependent biotransformation processes in *Escherichia coli*. Appl Microbiol Biotechnol.

[23] Woolston BM, King JR, Reiter M, Van Hove B, Stephanopoulos G (2018). Improving formaldehyde consumption drives methanol assimilation in engineered *E. coli*. Nat Commun.

[24] Gonzalez JE, Bennett RK, Papoutsakis ET, Antoniewicz MR (2018). Methanol assimilation in *Escherichia coli* is improved by co-utilization of threonine and deletion of leucine-responsive regulatory protein. Metab Eng.

[25] Price JV, Chen L, Whitaker WB, Papoutsakis E, Chen W (2016). Scaffoldless engineered enzyme assembly for enhanced methanol utilization. Proc Natl Acad Sci USA.

[26] Ma W, Cao W, Zhang B, Chen K, Liu Q, Li Y (2015). Engineering a pyridoxal 5′-phosphate supply for cadaverine production by using *Escherichia coli* whole-cell biocatalysis. Sci Rep.

[27] Nash T (1953). The colorimetric estimation of formaldehyde by means of the Hantzsch reaction. Biochem J.

