# Supplementary material for: Methanol fermentation increases the production of NAD(P)H-dependent chemicals in synthetic methylotrophic Escherichia coli
Source: Biotechnol Biofuels. 2019 Jan 21;12:17. doi: 10.1186/s13068-019-1356-4 (PMC6340170; doi:10.1186/s13068-019-1356-4)

**Original Research Paper**

**Methanol fermentation increases the production of NAD(P)H-dependent chemicals in synthetic methylotrophic *Escherichia coli***

Xin Wang^1^

Email: [xinwang1988@njtech.edu.cn](mailto:xinwang1988@njtech.edu.cn)

Xuelin Wang^1^

Email: [18305197928@163.com](mailto:18305197928@163.com)

Xiaolu Lu^1^

Email: [lxl87823629@126.com](mailto:lxl87823629@126.com)

Kequan Chen^1*^

Email: [kqchen@njtech.edu.cn](mailto:kqchen@njtech.edu.cn)

Pingkai Ouyang^1^

Email:ouyangpk@njtech.edu.cn

1. State Key Laboratory of Materials-Oriented Chemical Engineering, College of Biotechnology and Pharmaceutical Engineering, Nanjing Tech University, Nanjing 211816, Jiangsu, China

*Corresponding authors: Kequan Chen

Email: kqchen@njtech.edu.cn

**Table S1 Strains and plasmids used in this work**

| **Strains and plasmids** | **Description** | | **References** | |
| --- | --- | --- | --- | --- |
| **Strains** |  | | |  |
| BL21(DE3) | F¯*ompT gal dcm rne131 lon hsd*S_B_ (r_B_-m_B_- ) λ(DE3) | | | Invitrogen |
| BL21/Δ*frmA* | *E. coli* BL21(DE3); Δ*frmA* | | | This study |
| BL21/MDH | *E. coli* BL21/Δ*frmA* harboring pETDuet-*Mdh2* | | | This study |
| BL21/Hps-Phi | *E. coli* BL21/Δ*frmA* harboring pETDuet-Hps-Phi | | | This study |
| BL21/ΔfrmA-Mdh-Hps-Phi | *E. coli* BL21/Δ*frmA* harboring pETDuet-Mdh2-Hps-Phi | | | This study |
| BL21/ΔfrmA-ACT-Mdh-Hps-Phi | *E. coli* BL21/Δ*frmA* harboring pETDuet-*ACT-Mdh2*-*Hps-Phi* | | | This study |
| BL21/ΔfrmA-NudF-Mdh-Hps-Phi | *E. coli* BL21/Δ*frmA* harboring pETDuet-*NudF-Mdh2*-*Hps-Phi* | | | This study |
| BL21/ΔfrmA-ML | *E. coli* BL21/Δ*frmA* harboring pETDuet-*NudF*-*Mdh2*-*Hps*-*Phi* and pCWJ-*dapA*-*dapB*-LysC^fbr^-*PPC* | | | This study |
| BL21/ΔfrmA-ML-POS5 | *E. coli* BL21/Δ*frmA* harboring pETDuet-*Mdh2*-*Hps*-*Phi-POS5* and pCWJ- *dapA*-*dapB*-LysC^fbr^-*PPC* | | | This study |
| **Plasmids** | |  | |  |
| **pETDuet** | f1 *ori*, *lac*I, Amp^R^, P_T7_ | | | This study |
| **PCWJ** | RSF *ori*, *lac*I, Cm^R^, P_trc_ | | | (Patel, 2008) |
| **pETDuet -*Mdh2*** | Amp^R^, *P_T7_*, *B. methanolicus Mdh2 and B. methanolicus*  *Hps-Phi* operon | | | This study |
| **pETDuet-ACT-Mdh2** | Amp^R^, *P_T7_*, *B. methanolicus Mdh2 and B. methanolicus*  *ACT* | | | This study |
| **pETDuet-Mdh2-Hps-Phi** | Amp^R^, *P_T7_*, *B. methanolicus Mdh2 and B. methanolicus*  *Hps-Phi* operon | | | This study |
| **pETDuet-nudF-Mdh2 -Hps-Phi** | Amp^R^, *P_T7_*, *E. coli NudF*, *B. methanolicus Mdh2 and B. methanolicus Hps-Phi* operon | | | This study |
| **pCWJ-*dapA*-*dapB*-LysC^fbr^-*PPC*** | Cm^R^, *P_Trc_*, *E. coli dapA*, *E. coli dapB*, *E. coli LysC^fbr^* and *E. coli* PPC | | | This study |
| **pETDuet-*Mdh2*-*Hps*-*Phi- POS5*** | Amp^R^, *P_T7_*, *B. methanolicus Mdh2, B. methanolicus*  *Hps-Phi* operon *and S. cerevisiae POS5* | | | This study |
| **pCas** | Amp^R^, *pSC101 ori*, *P_cas_-cas9, P*_araB_-*Red* | | | Addgene |
| **pTarget** | *Smr^R^*, *pMB1 ori, , P_pJ23119_* | | | Addgene |
| **pTargetF-frmA** | *Smr^R^*, *pMB1*, *P_pJ23119_*_-_*sgRNA-frmA* | | | This study |

**Table S2 Primers used in this study**

| Name | Sequences (5′–3′) |
| --- | --- |
| Primer 1 | CGAGCTCATGCTTAAGCCAGACAACCTG |
| Primer 2 | ACGCGTCGACTTATGCCCACTCATTTTTTAACGCTTG |
| Primer 3 | GGAATTCCATATGATGAGTACGTTGGATTCACATTCC |
| Primer 4 | CCGCTCGAGTTAATCATTATCAGTCTGTCTCTTGG |
| Primer 5 | CCTGGAATAACTCGATAATACGACTCACTATAGGGGAATTGTGAGCG |
| Primer 6 | CTTTACCAGACTCGATTAATCATTATCAGTCTGTCTCTTGGTCAGC |
| Primer 7 | GGACTAGTTTGACAATTAATCATCCGGCTCG |
| Primer 8 | CATGCCATGGTTAGTGGTGGTGGTGGTGGTGCTCAAACAAATTACTATGCAGTTTTTG |
| Target-frmA-F | CCCGTTGTAAGAAAAACGGGGTTTTAGAGCTAGAAATAGC |
| Target-frmA-R | CCCGTTTTTCTTACAACGGGACTAGTATTATACCTAGGACTGAGC |
| frmA-cas-F1 | TTATCAGGCCTACGGCTTACGG |
| frmA-cas-R1 | AAGAGCGAGAGTTTCCCGCAGGTTTACCCCGT |
| frmA-cas-F2 | CTGCGGGAAACTCTCGCTCTTCCTCAATATGGTAATAGA |
| frmA-cas-R2 | CAACCCGAACGGGTATTGCC |

**Figure S1.** The growth of BL21/ΔfrmA-NudF-Mdh2-Hps-Phi under the different concentration of methanol.


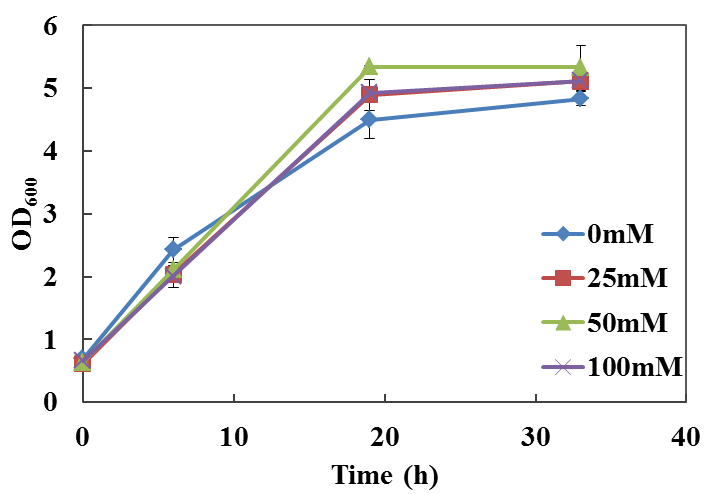

Supplement: Supplementary file 1 — Additional file 1: Table S1. Strains and plasmids used in this work. Table S2. Primers used in this study. Figure S1. The growth of BL21/ΔfrmA-NudF-Mdh2-Hps-Phi under the different concentration of methanol. [file 13068_2019_1356_MOESM1_ESM.docx]
